# Vitamin D-Binding Protein and the Free Hormone Hypothesis for Vitamin D in Bio-Naïve Patients with Psoriasis

**DOI:** 10.3390/ijms23031302

**Published:** 2022-01-24

**Authors:** Maria Siekkeri Vandikas, Kerstin Landin-Wilhelmsen, Martin Gillstedt, Amra Osmancevic

**Affiliations:** 1 Department of Dermatology and Venereology, Institute of Clinical Sciences, Sahlgrenska Academy, University of Gothenburg, SE-413 45 Gothenburg, Sweden; martin.gillstedt@vgregion.se (M.G.); amra.osmancevic@vgregion.se (A.O.); 2 Department of Internal Medicine and Clinical Nutrition, Institute of Medicine, Sahlgrenska Academy, University of Gothenburg, SE-413 45 Gothenburg, Sweden; kerstin.landin-wilhelmsen@gu.se; 3 Department of Dermatology and Venereology, Sahlgrenska University Hospital, Region Västra Götaland, SE-413 45 Gothenburg, Sweden

**Keywords:** vitamin D-binding protein, serum biomarker, psoriasis, vitamin D, 25-hydroxyvitamin D, free vitamin D

## Abstract

High levels of vitamin D-binding protein (DBP) have been reported in patients with psoriasis and the possibility of DBP as a marker of inflammation has been discussed. Furthermore, high DBP levels might negatively affect free 25(OH)D concentrations. According to the free hormone hypothesis, only the free fraction of a steroid hormone is capable of exerting biological action. Thus, free 25(OH)D level could be a better biomarker of vitamin D status than total 25(OH)D level. The objectives of this study were to identify the strongest determinants for DBP levels and to test the free hormone hypothesis for vitamin D in psoriasis. Additionally, we also aimed to investigate correlations between directly measured free 25(OH)D levels in serum and psoriasis disease severity compared to total 25(OH)D levels. This was a retrospective cross-sectional study including 40 bio-naïve patients with mild to severe plaque psoriasis. Psoriasis disease severity was evaluated using high sensitivity C-reactive protein (hsCRP), Psoriasis Area Severity Index (PASI) and visual analogue scale (VAS). Vitamin D metabolites including directly measured free 25(OH)D and serum DBP levels were measured. DBP levels were higher in patients with self-reported arthropathy than those without irrespective of confounding factors like sex, age and body weight. Total and free 25(OH)D levels correlated well (ρ = 0.77, *p* < 0.0001) and both were inversely correlated to intact parathyroid hormone (iPTH) (ρ = −0.33, *p* = 0.038 for total 25(OH)D and ρ = −0.40, *p* = 0.010 for free 25(OH)D). Only total 25(OH)D correlated to serum calcium levels (ρ = 0.32, *p* = 0.047). No correlations between any of the vitamin D metabolites and psoriasis disease severity were observed. In conclusion, DBP might be a new inflammatory biomarker in psoriasis, especially in psoriatic arthritis. Total 25(OH)D was a reliable measure for vitamin D status in this psoriasis cohort. However, evaluation of free 25(OH)D in patients with psoriatic disease and multiple co-morbidities and/or ongoing biologic treatment should be considered.

## 1. Introduction

The importance of vitamin D in psoriasis is controversial with the exception of effective local treatment with vitamin D analogues [1]. The association of vitamin D deficiency with disease severity and the benefit of vitamin D oral supplementation are disputed since studies have not yielded consistent results [2,3]. A possible reason for this inconsistency might be the use of an inadequate biomarker to measure vitamin D status in the blood. The internationally accepted metabolite that is used to estimate an individual’s vitamin D status is the total amount of the prohormone 25-hydroxyvitamin D (25(OH)D), which is measured in serum. Total 25(OH)D includes the part of 25(OH)D that is tightly bound to vitamin D-binding protein (DBP) (≈85%), the part that is loosely bound to albumin (≈15%) and the minuscule fraction that is free (<0.1%) [4].

The free hormone hypothesis is applicable for all steroid hormones and according to this hypothesis, the fraction of the hormone that is bound on its protein carriers is inactive. Only the free fraction (unbound) is able to passively cross the cell membrane, become hydroxylated to the active metabolite (1,25 (OH)_2_D) and exert biological action [5]. The free hormone hypothesis is applied in clinical praxis for thyroid and sex hormones, where the free levels of these hormones are measured, but is not clinically applied for vitamin D.

DBP determines the bioavailability of 25(OH)D in two different ways: (1) by delivering 25(OH)D directly into the cells via endocytosis when the megalin-cubilin receptor is expressed (in kidney, placenta, thyroid and mammary cells) and (2) by regulating the fraction of free 25(OH)D that is able to enter the cells that do not express megalin-cubilin receptors (most of the body’s tissues and cells) through passive penetration of the cell membrane [6].

The amount of free 25(OH)D depends on both the serum levels of DBP (high DBP levels bind more 25(OH)D leaving less circulating 25(OH)D as free) as well as the polymorphism of DBP, which affects the strength of the binding affinity for vitamin D metabolites [7]. Thus, the equilibrium between total and free 25(OH)D can vary [5]. Until recently, mathematical models were used to calculate levels of free 25(OH)D [8]. With the arrival of a commercial method using a two-step ELISA to directly measure levels of free 25(OH)D, investigations have shown that the calculated levels of free 25(OH)D are much higher than the directly measured levels in serum [8]. Furthermore, in some studies it was proposed that the level of free 25(OH)D and not total 25(OH)D was a better marker of the biological activity of vitamin D [9,10].

In a previous study, higher levels of DBP were observed in patients with psoriasis compared to healthy controls, which led to the hypothesis that total 25(OH)D might not be a reliable marker for vitamin D status since the amount of free 25(OH)D may be negatively affected by the high DBP levels [11]. In another study it was found that DBP levels were higher in subjects with psoriasis who reported arthropathy compared to those who did not [12]. Thus, the possible role of DBP in the systemic psoriatic inflammatory process was proposed, taking into consideration the biologic functions of DBP apart from vitamin D transportation, that is, actin scavenging, enhancing chemotaxis and macrophage activation [13,14].

In this study, the strongest determinants for the serum levels of DBP in psoriasis were investigated. Furthermore, the free hormone hypothesis was tested for its applicability to psoriasis. We hypothesized that there would be a stronger correlation between psoriasis disease severity and directly measured free 25(OH)D levels compared to total 25(OH)D levels in serum. Psoriasis disease severity was measured using high sensitivity C-reactive protein (hsCRP), Psoriasis Area Severity Index (PASI) and visual analogue scale (VAS). Directly measured free 25(OH)D and calculated free 25(OH)D levels were compared.

## 2. Results

### 2.1. Demographics

Forty adult bio-naïve patients with chronic plaque psoriasis (25 men and 15 women) were included. The mean age of the subjects was 47 years and the mean duration of disease was 24 years. Demographic data of the subjects including disease severity, sun habits, skin type, medication and comorbidities that might constitute confounding factors for vitamin D and DBP are presented in Table 1.

Sufficient 25(OH)D levels (25(OH)D ≥ 75 nmol/L) were observed in 15 subjects (38%), whereas 25 subjects (62%) had insufficient levels (<75 nmol/L). Detailed biochemical data for the 40 patients are shown in Table 2.

### 2.2. Seasonal Variation of Vitamin D Metabolites and Serum DBP

No difference in total 25(OH)D levels was noted between those who were recruited in winter compared to those recruited in summer (63.2 nmol/L versus 83.1 nmol/L, *p* = 0.10). On the other hand, both directly measured free 25(OH)D and bioavailable 25(OH)D levels were higher in those recruited during summer than winter (12.7 pmol/L and 4.6 nmol/L versus 9.6 pmol/L and 3.5 nmol/L, *p* = 0.013 and *p* = 0.030, respectively). No seasonal variation of serum DBP level was observed.

### 2.3. Serum DBP Levels in Relation to Demographics, Comorbidities, Psoriasis Severity, Vitamin D Metabolites and Other Parameters

The association between DBP and different variables is shown in Table 3. There were no differences in DBP levels between men and women and smokers versus non-smokers. There were no correlations between DBP and age, body mass index (BMI) and disease duration. No correlation between DBP and psoriasis disease severity (measured using hsCRP levels, PASI and VAS) was found.

A positive correlation was observed between levels of DBP and total 25(OH)D as well as directly measured free 25(OH)D but not between DBP and 1,25(OH)2D and intact parathyroid hormone (iPTH) respectively (Table 3).

**Figure 1:**
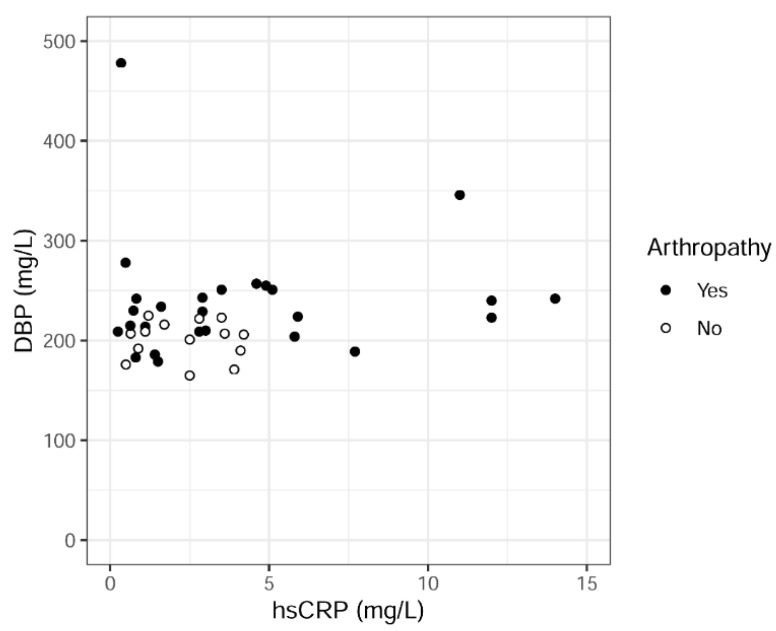
Association between vitamin D-binding protein (DBP) and high sensitivity C-reactive protein (hsCRP) levels in serum after stratifying subjects to those with self-reported arthropathy and those without. There was no significant correlation between levels of DBP and hsCRP (*p* = 0.32) but DBP levels were significantly higher in the subjects with self-reported arthropathy (*p* = 0.002) compared to those without.

Higher DBP levels were found in patients with self-reported arthropathy compared to those without (mean value 239 μg/mL versus 201 μg/mL, *p* = 0.002) but there was no significant correlation between DBP and hsCRP (*p* = 0.32) (Figure 1). When stratifying with respect to arthropathy (arthropathy: yes or no), no differences were observed between the two groups in sex, age, BMI, smoking habits, prevalence of diabetes and medication that might affect DBP levels (i.e., aspirin, hormonal contraception).

### 2.4. Association between Total and Directly Measured Free 25(OH)D Serum Levels

**Figure 2:**
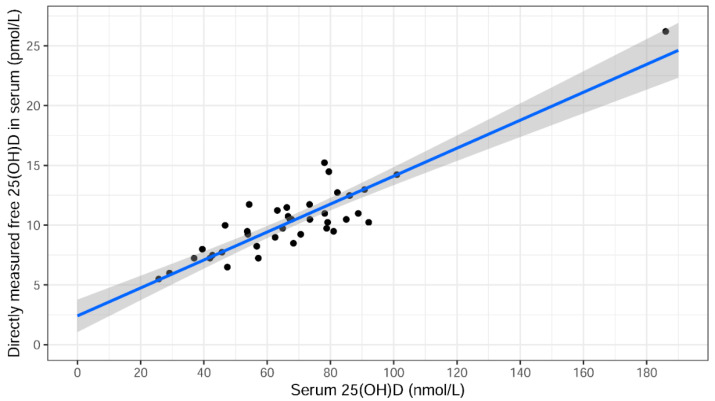
Association between directly measured free 25(OH)D and total 25(OH)D serum levels. The fitted line has the equation y = 2.41 + 0.117x. The grey area denotes the 95% confidence interval curves for the fitted line.

Total 25(OH)D levels correlated well with directly measured free 25(OH)D levels in serum as shown in the univariate linear regression model in Figure 2. The fitted line has the equation y = 2.41 + 0.117x and the grey area denotes the 95% confidence interval curves for the fitted line.

### 2.5. Association between Directly Measured Free 25(OH)D and Calculated Free 25(OH)D Serum Levels According to Bikle

**Figure 3:**
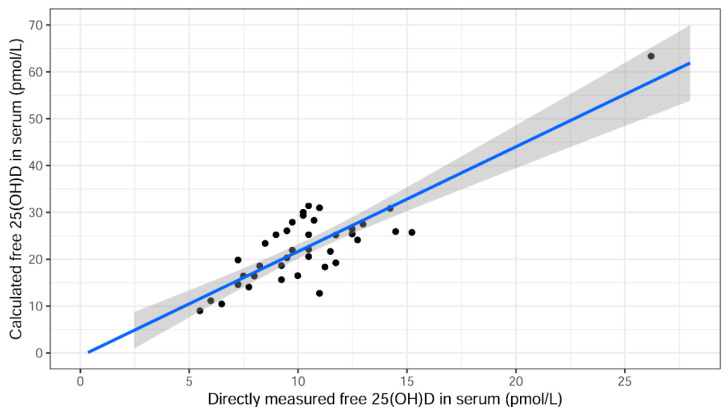
Association between calculated free and directly measured free 25(OH)D serum levels according to Bikle [15]. The equation for the fitted line is y = −0.714 + 2.24x.

Directly measured free and calculated free 25(OH)D serum levels, according to Bikle [15], correlated well but the levels of calculated free 25(OH)D were higher as shown in the univariate linear regression model in Figure 3. The equation for the line was y = −0.714 + 2.24x.

### 2.6. Total 25(OH)D, Directly Measured Free 25(OH)D and Bioavailable 25(OH)D Levels in Serum in Association with Psoriasis Disease Severity (Free Hormone Hypothesis)

No correlation was shown between any of the vitamin D metabolites and hsCRP levels, PASI and VAS results, respectively.

There were no correlations between 25(OH)D serum levels and hsCRP, PASI and VAS results even when stratifying subjects by 25(OH)D sufficiency (25(OH)D ≥ 75 nmol/L versus those with 25(OH)D < 75 nmol/L).

To test whether free 25(OH)D serum levels could be a more sensitive marker for the disease severity than serum levels of total 25(OH)D, subjects were categorized into those with free 25(OH)D ≥ 10.2 pmol/L (median value), *n* = 19, and those with free 25(OH)D < 10.2 pmol/L, *n* = 21. No differences in hsCRP, PASI and VAS results were noticed between the two groups.

### 2.7. Other Information

Free, total and bioavailable 25(OH)D serum levels correlated negatively to iPTH (ρ = −0.40, *p* = 0.010 for free 25(OH)D, ρ = −0.33, *p* = 0.038 for total 25(OH)D and ρ = −0.39, *p* = 0.014 for bioavailable 25(OH)D).

Albumin corrected calcium correlated positively with total 25(OH)D levels (ρ = 0.32, *p* = 0.047) but not with directly measured free 25(OH)D levels (ρ = 0.30, *p* = 0.063).

Alkaline phosphatase (ALP) levels were within normal values.

**Figure 4:**
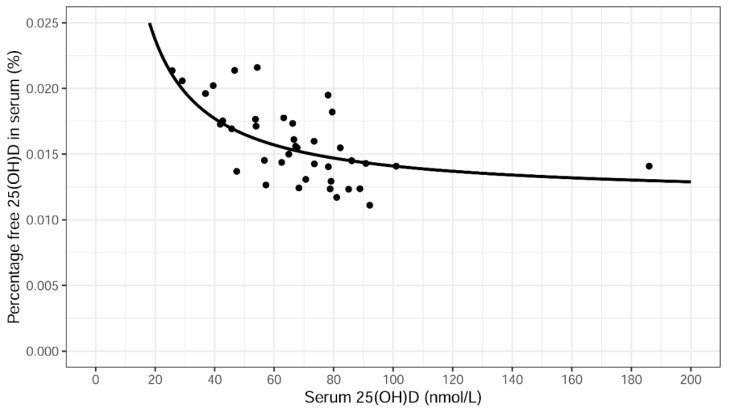
Association between the percentage free 25(OH)D and total 25(OH)D levels in serum. The fitted curve is based on the line in Figure 2 and has the equation y = 0.241/x + 0.0117.

Serum levels of hsCRP correlated positively with BMI (ρ = 0.52, *p* < 0.001). No correlations were found between any of the vitamin D metabolites and BMI. A negative association was found between levels of total 25(OH)D and the percentage of free 25(OH)D (ρ = −0.66, *p* < 0.001) in serum and is shown using a univariate linear regression in Figure 4. The fitted curve is based on the line in Figure 2 divided by x and after changing the units in order to get the percentage of free 25(OH)D. It has the equation y = 0.241/x + 0.0117.

## 3. Discussion

The present study confirmed that in patients with psoriasis, DBP levels were higher in those with self-reported arthropathy compared to those without, as shown before [12]. This finding could not be explained by BMI, age or other known confounding factors for high DBP levels such as female sex, aspirin use, diabetes, smoking, thyroid disease and use of hormonal contraception [16,17]. DBP is a multifunctional protein with clinical importance and has a possible role in the pathogenesis or susceptibility of different diseases like cancer, cardiovascular, autoimmune and inflammatory diseases [18,19,20,21]. There are unmet needs regarding diagnostic biomarkers for early detection of psoriatic arthritis (PsA). The ClASsification for Psoriatic ARthritis (CASPAR) criteria that accurately assist the diagnosis of PsA have the disadvantage that they do not detect early PsA due to the low prevalence of radiographic damage in early disease [22]. It would therefore be interesting to evaluate DBP serum levels in prospective studies in patients with skin psoriasis to investigate whether DBP concentration could have a predictive role in the later development of PsA and thus could be used as an early diagnostic biomarker. In this study, contrary to other studies [23,24], DBP levels were not found to be associated with hsCRP levels. This might be explained by the small study sample but nevertheless hsCRP is not always the best marker for systemic inflammation in psoriasis and PsA [25]. DBP level was not associated with any other variables of psoriasis disease severity (PASI and VAS) either. Furthermore, DBP level in serum was independent of sex, age, smoking habits, season of inclusion and BMI.

To the best of our knowledge this is the first time the free hormone hypothesis in vitamin D has been tested in bio-naïve patients with chronic plaque psoriasis and considering DBP levels. The data show that in this psoriasis cohort, total 25(OH)D levels correlated strongly with directly measured free 25(OH)D levels in serum and both were negatively correlated to iPTH, an expected biological effect of vitamin D. Furthermore, only total 25(OH)D correlated to serum calcium. The above information, put together, shows that total 25(OH)D levels could be used as a reliable marker for patients’ vitamin D status. This leads to the conclusion that the internationally acceptable marker for vitamin D status, total 25(OH)D serum levels, is reliable and most probably not an explanatory factor for the conflicting results in the literature regarding vitamin D and psoriasis.

However, one must consider that this cohort consisted of a relatively young (mean age 47 years) and quite homogeneous group of patients with no serious comorbidities like kidney or liver disease or other autoinflammatory diseases. Diseases and medications believed to affect DBP concentration (i.e., diabetes, hormonal contraception and aspirin use) were not overrepresented in this group. This might also explain the fact that DBP levels were within the normal range [23] and no disruption in the equilibrium between bound and free 25(OH)D had occurred. The free hormone hypothesis needs to be tested in larger populations of patients with psoriasis and a variety of comorbidities as well as ongoing treatment.

No correlations could be found between free and/or total 25(OH)D serum levels and disease severity measured using hsCRP, PASI and VAS. However, it would be highly interesting to examine whether vitamin D levels are low at the onset of psoriasis. Low vitamin D levels might be involved in the pathogenesis of psoriasis in the early stages of the disease when the disruption between the Th2 and Th1/Th17/Th22 immune response occurs. After being diagnosed with psoriasis, sun habits and consequently vitamin D levels might change in these patients as heliotherapy has a known fast and good effect on psoriatic skin inflammation [26,27]. Even ultraviolet B phototherapy that is given as a first line treatment can positively affect vitamin D levels [26].

To date, there are three published studies evaluating directly measured free 25(OH)D serum levels in patients with psoriasis undergoing biologic therapy where the results were compared with healthy controls [12,28,29]. It is known that biologic treatment and especially tumor necrosis factor (TNF)-α inhibitor treatment may negatively affect total 25(OH)D levels in serum [30]. In two of these studies [28,29], patients with psoriasis had lower amounts of free 25(OH)D compared to healthy controls and in one of the studies [28], free 25(OH)D levels were lower despite similar total 25(OH)D levels between the two groups, speaking in favor of the free hormone hypothesis and that free 25(OH)D level might be a more accurate marker of vitamin D status than total 25(OH)D level. However, DBP was not measured in these two studies.

Calculated free 25(OH)D concentrations were higher than directly measured free 25(OH)D concentrations, as shown before, which further confirms that the formulas used to calculate free 25(OH)D levels are not very accurate [31]. An interesting observation was that the percentage of free 25(OH)D is inversely correlated to total 25(OH)D levels, which implies that an adjustment mechanism in the body exists for higher release of free 25(OH)D, mainly in those with low total 25(OH)D levels, as earlier reported [23].

Limitations of this study include: (1) that it was a cross-sectional study without healthy controls, (2) no clinical data were collected for specifying the self-reported arthropathy, (3) the method used for measuring total 25(OH)D levels is not the gold standard LC-MS/MS but still a validated method [32], (4) the method used for measuring DBP levels might underestimate the amount of DBP in patients with African ancestry [7] but this can be overcome by the fact that all subjects were Caucasian, (5) recruitment occurred in various periods of sunlight (winter, summer) and (6) the small sample size. Therefore, the obtained data should be treated as a preliminary study with limited conclusions.

## 4. Materials and Methods

### 4.1. Study Design, Setting and Participants

This was a retrospective cross-sectional study including 40 Caucasian, bio-naïve patients with chronic plaque psoriasis who were followed up at the outpatient clinic of the Department of Dermatology at Sahlgrenska University Hospital, Gothenburg, Sweden, between 2013 and 2017.

Subjects were ≥18 years with mild to severe plaque psoriasis. All subjects were examined by an experienced dermatologist who could confirm the diagnosis clinically. Exclusion criteria were: pregnancy/lactation or plans for pregnancy; ongoing other severe chronic or systemic disease, e.g., liver, kidney, cancer or infectious disease; treatment with oral steroids or other immunosuppressive/anti-inflammatory drugs and antibiotic treatment; ongoing sunbed use or sunbed use during the last 4 weeks; and sun holiday for the past 4 weeks. A questionnaire was completed including medical history, medication, dietary supplements, sun habits and other lifestyle variables that could affect vitamin D status, DBP and inflammation. PASI was used for scoring the severity of psoriasis in the skin. VAS was used as a simple method for evaluating self-rated psoriasis activity, where subjects mark the intensity of their symptoms on a 10 cm long line (0 means no complaints and 10 worst complaints). VAS has previously been used in assessing psoriasis severity and has shown good correlation to PASI and the Dermatology Life Quality Index (DLQI) [17].

The skin type according to Fitzpatrick [15] was defined. BMI was calculated using body weight and height. BMI ≥ 30 kg/m^2^ was classified as obese. 25(OH)D serum concentration ≥75 nmol/L was defined as sufficient according to the Endocrine Society [16].

Patients recruited from October to March, when the ultraviolet (UV) index in Gothenburg is <3, were classified as recruited in winter and those recruited from April to September, when the UV index is ≥3 and thus vitamin D production in the skin is possible, were classified as recruited in summer.

### 4.2. Blood Samples and Analyses

All samples were drawn in the morning, no fasting was required. The laboratory methods used are described in detail in a previous article [12]. Total 25(OH)D (25(OH)D_2_ and 25(OH)D_3_) levels were analyzed with an electrochemiluminiscence immunoassay (ECLIA) using the Elecsys Vitamin D Total II assay, on a Cobas 8000 Roche instrument (Roche Diagnostics Scandinavia AB, Tokyo, Japan).

Free 25(OH)D concentrations were measured with a two-step immunosorbent assay (ELISA) performed using a commercial kit (Future diagnostics B.V., Wijchen, The Netherlands).

1,25(OH)_2_D was analyzed with an automated chemiluminescence immunoassay (CLIA) with an IDS-iSYS instrument (IDS, Boldon, UK).

DBP was analyzed with a monoclonal ELISA (R&D systems, Minneapolis, MN, USA).

Serum iPTH was analyzed with ECLIA (electro chemiluminescence immunoassay) with an Elecsys PTH STAT, article number: 04892470190 (Roche Diagnostics Scandinavia AB, Tokyo, Japan).

The serum levels of hsCRP, creatinine, albumin, calcium and ALP were analyzed with standardized laboratory techniques on a Cobas Roche instrument (Roche Diagnostics Scandinavia AB, Tokyo, Japan).

Corrected calcium was calculated using the formula:

serum calcium + 0.02 ∗ (40 − albumin).

### 4.3. Calculation of Free 25(OH)D, Bioavailable 25(OH)D and the Percentage of Free 25(OH)D

The calculation of free and bioavailable 25(OH)D (the sum of the amount of 25(OH)D that is loosely bound to albumin and the directly measured free fraction) was done using the equations by Bikle [18] and as described in detail before [11].

In order to calculate the percentage of free 25(OH)D, the concentration of free 25(OHD) that was initially measured in pg/mL was converted to pmoL/L using the formula 1 pg/mL = 2.496 pmoL/L. The percentage free 25(OH)D was then calculated as free 25(OH)D divided by total 25(OH)D.

### 4.4. Statistical Analyses

All data were analyzed using R version 3.5.3 (The R Foundation for Statistical Computing, Vienna, Austria). Simple descriptive statistics were applied. We used Spearman’s correlation test in order to test for univariate correlations like the correlation between total and directly measured free 25(OH)D, correlations between vitamin D metabolites and calcium as well as iPTH and psoriasis disease severity (hsCRP, PASI and VAS), respectively. Spearman’s correlation test was also used to test the correlation between hsCRP and BMI. Wilcoxon’s rank sum test was used for two sample tests for example to test for a difference in DBP for those with and without arthropathy as well as to compare the levels of vitamin D metabolites and DBP between those recruited in summer and those recruited in winter. Fisher’s exact test was used to compare proportions for example when stratifying the patients with respect to arthropathy (arthropathy: yes or no), to test for differences between the two groups in sex, age, BMI, smoking habits, prevalence of diabetes and medication.

Linear multiple regression models were used with DBP as the dependent variable, the variables in Table 3 as independent variables, and age and sex included as covariates. Outliers were removed for DBP and 25(OH)D in the appropriate regression models. Linear regression was also used to correlate directly measured free 25(OH)D with total 25(OH)D and also directly measured free 25(OH)D versus calculated free 25(OH)D. Goodness of fit tests were used by testing the residuals in the linear regression models with the Shapiro–Wilk test for normality. All tests were two-sided and *p* < 0.05 was considered as statistically significant.

## 5. Conclusions

DBP might be a new inflammatory biomarker in psoriasis especially in patients with concurrent arthritis and this finding warrants further investigation. In this psoriasis cohort, total 25(OH)D serum levels correlated well with free 25(OH)D levels in serum and seemed to be an accurate marker for vitamin D status. No associations were found between free or total 25(OH)D serum levels and psoriasis disease severity. However, it would be interesting to analyze serum vitamin D levels in new-onset psoriasis.

## Figures and Tables

**Table 1:** Demographic data for all the subjects with psoriasis. SD = Standard Deviation.

	Patients with Psoriasis						
	*n* = 40						
	Median	IQR	Mean	SD	95% CI		*n*
					Lower	Upper	
Age (Years)							
Men	46	[41–58]	48	13	43	54	25
Women	48	[29–58]	46	17	37	55	15
All	47	[37–59]	47	15	43	52	40
Duration of psoriasis (years)	25	[12–33]	24	14	19	28	39
PASI ^†^	9.7	[7.8–13.9]	11.2	4.9	9.7	12.8	40
Mild psoriasis (<5)	1/40 (3%)						
Moderate psoriasis (≥5, <10)	19/40 (48%)						
Severe psoriasis (≥10)	20/40 (50%)			s			
	**Median**	**IQR**	**Mean**	**SD**	**95% CI**		***n***
					**Lower**	**Upper**	
Total hours spent outdoors during summer	5.0	[3.5–8.0]	5.5	3.0	4.5	6.5	37
Fish meals/week	1.0	[1.0–2.0]	1.6	0.8	1.3	1.9	35
	**II**	**III**	**IV**				
Skintype (*n*)	15	24	1				
Proportion	37.5%	60.0%	2.5%				
	***n***	**%**					
Self-reported arthropathy	25	64%					
Current smokers	17	43%					
Antidyslipidemic use	5	13%					
Antihypertensive use	6	15%					
Antidiabetic use	2	5%					
Antidepressant use	5	13%					
Painkiller use	6	15%					
Hypothyroidism medication	2	5%					
Hormonal contraception	1	3%					
Aspirin	1	3%					
Obesity (BMI ^‡^ ≥ 30 kg/m^2^)	9	23%					

^†^ PASI = Psoriasis Area Severity Index. ^‡^ BMI = Body Mass Index.

**Table 2:** Biochemical data and the calculated vitamin D metabolites (calculated free 25(OHD), bioavailable 25(OH)D and the percentage free 25(OH)D) levels in serum. SD = Standard Deviation.

	Patients with Psoriasis						
	*n* = 40						
	Median	IQR	Mean	SD	95% CI		*n*
					Lower	Upper	
Serum 25(OH)D (nmol/L)	67	[54–80]	69	26	60	77	40
Directly measured free 25(OH)D in serum (pmol/L)	10.2	[8.4–11.5]	10.4	3.4	9.3	11.5	40
Calculated free 25(OH)D in serum (pmol/L)	22.0	[16.5–26.2]	22.6	9.0	19.8	25.5	40
Bioavailable 25(OH)D in serum (nmol/L)	3.7	[2.9–4.2]	3.8	1.2	3.4	4.2	40
Percentage free 25(OH)D in serum (%)	0.015	[0.014–0.018]	0.016	0.003	0.015	0.017	40
Serum 1,25(OH)_2_D (pmol/L)	89	[66–107]	95	43	81	108	40
PTH ^†^ (pmol/L)	3.3	[2.9–4.1]	3.7	1.3	3.2	4.1	40
DBP ^‡^ (mg/L)	216	[203–241]	226	52	209	243	40
hsCRP ^§^ (mg/L)	2.8	[1.0–4.3]	3.5	3.5	2.4	4.6	40
Albumin (g/L)	40.0	[39.0–42.0]	40.4	2.86	39.4	41.3	40
Creatinine (µmol/L)	78	[68–89]	78	15	73	83	40
Calcium (mmol/L)	2.41	[2.34–2.46]	2.41	0.086	2.38	2.44	40
ALP ^#^ (µkat/L)	1.20	[1.00–1.43]	1.27	0.43	1.13	1.41	40

^†^ iPTH = intact parathyroid hormone. ^‡^ DBP = vitamin D-binding protein. ^§^ hsCRP = high sensitivity C-reactive protein. ^#^ ALP = alkaline phosphatase.

**Table 3:** Linear regression tests with vitamin D-binding protein (DBP) as the dependent variable adjusting for age and sex.

Independent Variable in Linear Regression with DBP as Dependent Variable	Regression Coefficient (95% CI)	Adjusted *p*-Value *
Demographics		
Age (years)	0.22 (−0.36, 0.80)	0.46 **
Women vs. Men	−9.3 (−27, 8.1)	0.30 ***
BMI ^†^ (kg/m^2^)	−0.43 (−2.3, 1.5)	0.66
Smoking (Yes vs. No)	−1.8 (−19, 16)	0.84
Arthropathy (Yes vs. No)	24 (7.6, 39)	0.007
Vitamin D metabolites		
Serum 25(OH)D (nmol/L)	0.60 (0.15, 1.1)	0.014
Directly measured free 25(OH)D in serum (pmol/L)	4.5 (0.81, 8.2)	0.023
Percentage free 25(OH)D in serum (%)	−2096 (−5051, 860)	0.17
Serum 1,25(OH)2D (pmol/L)	0.21 (−0.0038, 0.42)	0.063
Other variables		
hsCRP ^‡^ (mg/L)	1.7 (−1.2, 4.5)	0.26
Disease duration (years)	−0.52 (−1.3, 0.28)	0.21
PASI ^#^	1.4 (−0.29, 3.1)	0.11
VAS ^€^	3.4 (−0.14, 6.9)	0.069
iPTH ^§^ (pmol/L)	−2.0 (−8.7, 4.7)	0.56

* Adjusted for age and sex; ** Adjusted for sex only; *** Adjusted for age only; ^†^ BMI = Body Mass Index; ^‡^ hsCRP = high sensitivity C-reactive protein; ^#^ PASI = Psoriasis Area Severity Index; ^€^ VAS = visual analogue scale; ^§^ iPTH = intact parathyroid hormone.

## Data Availability

The data presented in this study are available on request from the corresponding author. The data are not publicly available due to ethical restrictions.
